# Psychiatric conditions in autistic adolescents: longitudinal stability from childhood and associated risk factors

**DOI:** 10.1007/s00787-022-02065-9

**Published:** 2022-08-17

**Authors:** Matthew J. Hollocks, Virginia Carter Leno, Susie Chandler, Pippa White, Isabel Yorke, Tony Charman, Andrew Pickles, Gillian Baird, Emily Simonoff

**Affiliations:** 1 Department of Child and Adolescent Psychiatry, Institute of Psychiatry, Psychology and Neuroscience, King’s College London, 16 De Crespigny Park, London, UK; 2 South London and Maudsley NHS Foundation Trust (SLaM), London, UK; 3 Department of Biostatistics and Health Informatics, Institute of Psychiatry, Psychology and Neuroscience, King’s College London, London, UK; 4 Department of Psychology, Institute of Psychiatry, Psychology and Neuroscience, King’s College London, London, UK; 5 Maudsley Biomedical Research Centre for Mental Health, London, UK; 6 Newcomen Centre, Evelina Children’s Hospital, Guys and St Thomas NHS Foundation Trust, London, UK

**Keywords:** Autism, Longitudinal, Comorbidity, Adolescence, QUEST

## Abstract

Autistic people experience high rates of co-occurring psychiatric diagnoses. Current prevalence estimates vary considerably due to an over-reliance on clinical cohorts and the longitudinal stability of diagnoses from childhood into adolescence is poorly understood. This study aims to provide prevalence rates of co-occurring DSM-5 psychiatric diagnosis for autistic adolescence and investigate, for the first time, the stability of diagnoses from childhood. Using a longitudinal stratified sample of autistic youth (*N* = 77; 13–17 years; 60% male), selected from a larger community-derived sample of those with pre-existing autism diagnoses (*N* = 277) weighted prevalence estimates of emotional (anxiety, depression), behavioural (oppositional and conduct disorders) and ADHD diagnoses were calculated based on semi-structured psychiatric interview. Prediction of adolescent psychiatric diagnosis based on childhood diagnostic status, sex, childhood IQ (both assessed at age 4–10 years) was tested. Emotional and behavioural disorders in adolescence were particularly prevalent, and significantly predicted by childhood disorder status. Attention-deficit/hyperactivity-disorder (ADHD) was prevalent but not predicted by childhood ADHD diagnosis. Neither sex nor childhood IQ predicted diagnostic outcomes. Autistic youth have high levels of co-occurring psychiatric conditions, which are broadly persistent across childhood and adolescence. Emotional disorders are particularly prevalent and remain persistent from childhood to adolescence. Greater diagnostic variability was found for ADHD with more adolescents moving across diagnostic thresholds.

**Supplementary Information:**

The online version contains supplementary material available at 10.1007/s00787-022-02065-9.

## Introduction

Autism spectrum disorder (hereafter, autism) is a neurodevelopmental condition characterised by difficulties with reciprocal social communication, restricted interests, repetitive behaviours and sensory anomalies [1]. In addition to these core features, it is recognised that up to 70% of autistic individuals have at least one co-occurring psychiatric diagnosis [2], with up to 50% having multiple additional diagnoses [3]. Meta-analytic evidence from studies across the lifespan has demonstrated high prevalence rates across common child and adolescent diagnoses, in particular attention-deficit hyperactivity disorder (ADHD), emotional disorders (including anxiety and depression) and behavioural disorders (including oppositional defiant disorder (ODD) and conduct disorder [3]). Despite clear evidence of high rates of psychiatric diagnosis across these domains in autistic youth, there remains significant heterogeneity within prevalence estimates due to reliance on clinical samples and diagnoses. No studies to date have investigated the stability of diagnoses over time and which factors predict the likelihood of meeting diagnostic criteria. Understanding which conditions are persistent over development is vital for guiding the delivery of interventions and clinical services.

### Stability of psychiatric conditions in autistic and non-autistic children and adolescents

In non-autistic child and adolescent populations, increasing age is generally associated with an increasing prevalence of emotional disorders (i.e., anxiety and depression) and obsessive–compulsive disorder (OCD), whilst rates of behavioural disorders and ADHD tend to decrease [4, 5]. In terms of stability, analyses of combined population-representative cohorts of non-autistic youth show the highest within-disorder continuity from childhood to adolescence for ADHD (Odds ratio [OR] = 28.42), followed by behavioural (conduct disorder/oppositional defiant disorder; ORs = 7.45, 6.16, respectively) and emotional disorders (anxiety/depression; ORs = 3.18, 3.33, respectively) [6]. There is limited evidence available from longitudinal studies which include samples of autistic people, but data from cross-sectional studies conducted in different age groups suggest similar patterns of change across childhood and adolescence for anxiety, depression and ADHD [3], but with little evidence available for behavioural disorders. Those studies which do exist have reported stability in symptoms, in both the short (12–16 years [7]) and long term (10–23 years [8]). However, there have yet to be longitudinal studies that report prevalence of diagnoses (as opposed to symptoms) across timepoints on the same sample of autistic youth. Other sources, particularly those from clinical samples, indicate instead that DSM emotional disorders are more stable than behavioural disorders between childhood and adolescence [9, 10].

### Predictors of psychiatric conditions in autistic children and adolescents

In addition to understanding overall prevalence of psychiatric diagnosis in autistic youth, it is important to know which children may be at high risk of experiencing additional psychiatric difficulties. In the literature on typically developing populations, sex and lower intellectual ability are considered risk factors for a range of psychiatric disorders (with males at greater risk of behavioural diagnoses and females’ emotional diagnoses) [11, 12]. In autism studies, female sex has been associated with greater symptoms [13] and prevalence of emotional disorders [14]. Intellectual ability has also shown to relate to the prevalence of psychiatric comorbidity in autism. Intellectual functioning is highly heterogeneous within autistic individuals, ranging from those with an intellectual disability (between 30 and 50% of cases [15, 16]) up to those with intelligence in the superior range. The relationships between IQ and symptoms of psychiatric comorbidity have been mixed. There is some evidence to indicate that higher IQ is associated with greater symptoms of anxiety [17] and depression [13], although this may in part be driven by difficulty measuring internalizing disorders in individuals with intellectual disability; both in terms of caregivers recognising and individual’s themselves communicating relevant symptoms. Conversely, others report that lower IQ in autistic children predicts increased symptoms of hyperactivity [7], separation anxiety in adolescence [18], and poorer mental health outcomes in adulthood [19].

Most studies testing putative risk factors for additional psychiatric difficulties in autistic youth rely on measures of psychiatric symptoms, usually reported by a caregiver, and selective samples (e.g., volunteer or clinic) which are unlikely to be representative of the wider autistic population. To build a more solid evidence base for the types of psychiatric difficulties likely to be experienced by autistic people at different points in their lifespan, and risk factors for developing additional psychiatric diagnoses, research using validated clinical assessments is necessary, as these are thought to be less impacted by rater biases. Those that have used in-depth psychiatric assessments in population-representative autism cohorts report few significant associations, including a lack of impact of IQ, in youth aged 10–14 years [2]. Correlates of psychiatric diagnoses were tested in the first wave of the current study when participants were aged between 4 and 10 years [20]. Here, older children and those with a higher IQ were more likely to have anxiety diagnosis, and male gender significantly predicted a greater prevalence of both ADHD and ODD.

The aim of this study is to provide prevalence estimates of co-occurring psychiatric diagnoses weighted back to a community-derived cohort of autistic adolescents, aged between 13 and 17 years, to provide population-based rates. We will also test whether the factors that were previously associated with psychiatric diagnosis in childhood (sex and IQ) in this cohort continue to predict diagnostic status longitudinally in adolescence. As this sample purposefully over-sampled females, it gives sufficient power to test for sex differences in psychiatric diagnosis. Finally, taking advantage of the longitudinal design, we explore the stability of diagnoses from childhood into adolescence in different types of psychiatric disorder by testing whether diagnostic status in childhood predicts status for the same disorder in adolescence.

## Methods

### Sample

**Fig. 1:**
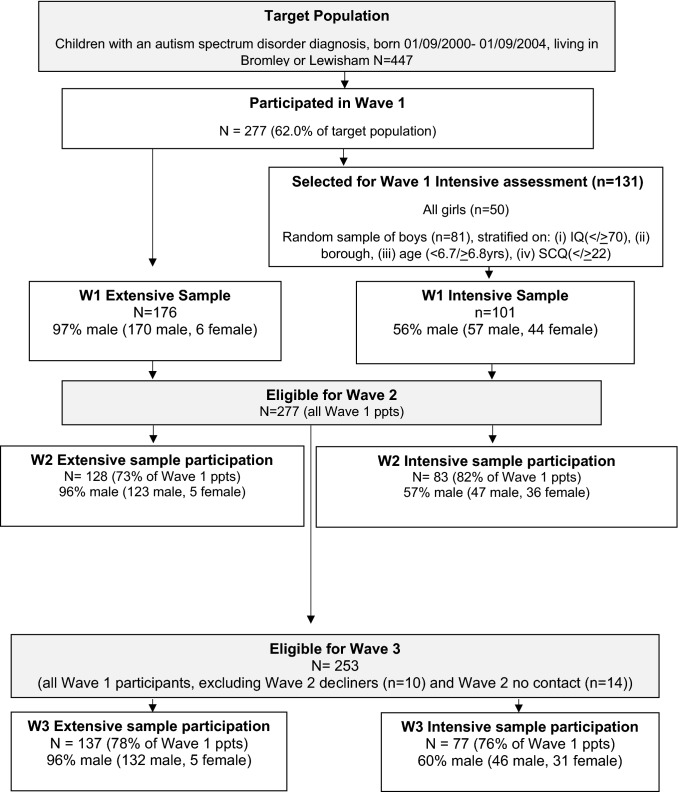
Overall sampling strategy and design of the QUEST follow-up study, Waves 1, 2 and 3

This study included participants from the QUEST study [20], a longitudinal community sample recruited at age 4–9 years (wave 1; *N* = 277) and followed-up throughout childhood as part of the IAMHealth project (http://iamhealthkcl.net/). See Supplementary Materials for a more detailed description and a flowchart of study recruitment and participation. Briefly, all children with an autism spectrum disorder diagnosis, born between September 2000 and September 2004, living in two London health districts were invited to participate (*n* = 447). Of that number response were received from 362 (81%), with 277 parents (62%) completing study questionnaires. Upon entry to the study, participants were split into an “intensively studied” (intensive; *n* = 101) and “extensively studied” group (extensive; *n* = 176), which was maintained throughout subsequent waves of data collection. Those in the extensive sample completed a range of clinical and questionnaire measures online, whilst the intensive sample completed additional in-person measures including the assessment of co-occurring psychiatric diagnoses at Wave 1 and Wave 3. Female participants were purposefully over-sampled to allow for sex comparisons, by inviting all females to join the intensive subsample. They were joined by a randomly selected group of males stratified to provide equal numbers on the following characteristics (1) IQ (< 70/ ≥ 70); (2) child’s age (4.5–6.7/6.8–9.9 years) and (3) Social Communication Questionnaire total score (< 21/ ≥ 22). Wave 2 of the study was conducted at ages 11–15 years (not reported on here), with Wave 3 being conducted at ages 13–17 years. See Fig. 1 for details.

### Measures

#### Co-occurring psychiatric diagnoses

*The Preschool Age Psychiatric Assessment (PAPA; Wave 1)* is a semi-structured, parent-reported interview for preschool children aged 2–5 years [21]. The PAPA assessed the presence of DSM-IV diagnoses in the three months prior to assessment. PAPA interviews were conducted by clinicians or research psychologists trained in its administration. Validation of interview administration and coding was performed by the trainer (from Duke University) at an early stage in the study. Specific coding issues were discussed on an ad hoc basis both with the US trainer and ES (who is author of the sister instrument, the CAPA). These included decisions about whether items met PAPA criteria for additional psychopathology or were more appropriately considered symptoms of autism. The detailed PAPA symptom criteria were used rigorously but in an agnostic fashion to endorse individual symptoms. Standardized algorithms previously developed and reported were used to determine diagnoses [22]. In line with the current DSM-5 criteria cases of ADHD were not excluded when in the presence of autism. PAPA data were collected from 101 participants.

*The Child and Adolescent Psychiatric Assessment (CAPA; Wave 3)* is the sister instrument of the PAPA designed to assess psychiatric diagnoses in children up to 17 years of age [23]. The CAPA was administered with parents who reported on their child’s symptoms over the preceding 3 months, which were used identify the presence of DSM-5 diagnoses. As with the PAPA, interviewers were trained in the standard administration and coding, and specific coding issues were discussed on an ad hoc basis with specific consideration around whether symptoms may be better understood in the context of autism. CAPA data were collected from 72 participants (Fig. 1).

#### Auxiliary variables

*Intellectual ability* IQ was measured at Wave 1 using one or more of the following tests, depending on the child’s age and developmental level: the Mullen Scales of Early Learning [24], the Wechsler Preschool and Primary Scale of Intelligence [25], and the Wechsler Intelligence Scale for Children [26]. Performance on these tests were converted to a standard score of representing full-scale IQ (FSIQ) with a mean of 100 and SD of 15.

*Social Communication Questionnaire-lifetime version* (SCQ) [27]. The SCQ, a 40-item questionnaire based on gold-standard’ autism diagnostic instruments, was used to assess the characteristics of autism at Wave 1.

*Developmental Behavior Checklist (DBC)* [28]. The DBC is a measure of behavioural and emotional problems typically used in children with developmental or intellectual difficulties. In this study, we used the parent-reported depressive, disruptive and hyperactivity behaviours sub-scales at Wave 1.

*Strengths and Difficulties Questionnaire (SDQ)* [29]*.* The SDQ is an emotional and behavioural screening questionnaire consisting of 25 questions, measuring 5 domains: (1) emotional symptoms; (2) conduct problems; (3) hyperactivity/inattention; (4) peer relationship problems; and (5) prosocial behaviour. This study used parent report at Wave 3 as a measure of emotional symptoms, conduct problems and hyperactivity/inattention. Due to skew, the conduct problems sub-scale was square root transformed.

### Statistical analyses

The analysis was structured as follows: (1) prevalence for each individual disorder was estimated; (2) we tested whether sex or intellectual ability measured at Wave 1 predicted Wave 3 diagnostic status; (3) we tested the likelihood of meeting threshold for a disorder at Wave 3 given diagnostic status at Wave 1 (i.e., diagnostic stability). Given the number of diagnoses once individual prevalence rates had been estimated, diagnoses were collapsed into three overarching categories for parsimony in tests of prediction by sex and intellectual ability and within-disorder stability. They consisted of emotional disorders (including anxiety disorders, depression and to be consistent with wave 1, OCD), behavioural disorders (ODD and conduct disorder), and ADHD.

To estimate prevalence and associations within the representative population, we made use of data from both the full community-derived sample of 277 participants and the selected sub-sample with full diagnostic assessment of mental health (intensive: *n* = 101 at Wave 1). This enabled us to use the information available on the larger extensive sample in the weighting adjustment to improve the accuracy of our prevalence estimates by reducing variability and decreasing potential bias in the prevalence estimates caused by non-response associated with the study design. This goes beyond the standard weighting approach using this auxiliary information to improve the accuracy of our weighted prevalence rates. To do this, we implemented an approach using a latent class model (where CAPA diagnosis was used as an error-free indicator of a diagnosis latent class) with auxiliary variables and covariates using the Stata gsem command for generalized structural equation modelling. This was followed, where required, by postestimation using the margins command. Latent class probabilities were allowed to be associated with sub-sample design variables from Wave 1 (sex, the district sampled from, FSIQ, age at recruitment, SCQ score) allowing for testing of the effects of these variables, and the margins command used to obtain rates of outcome at the estimated population means of these design variables. This approach also adjusts for non-designed attrition associated with both the auxiliary variables and the covariates [30].

To improve the power for the analysis testing longitudinal stability of diagnostic status, we included the DBC (Wave 1) and SDQ (Wave 3) that were available on the larger sample (*n* = 277) as additional error-prone auxiliary indicators of the corresponding CAPA diagnoses (see Supplementary Fig. 2) within each symptom domain (i.e., DBC depression and SDQ emotional problems in the emotional disorders model, DBC disruptive and SDQ conduct problems subscales in the behavioural disorders model, DBC hyperactivity and SDQ hyperactivity subscales in the ADHD model). Wave 1 social economic status (SES), measured using the 2007 English Indices of Multiple Deprivation (Department for Communities and Local Government, 2007) was used instead of district in these models as the binary district variable led to issues with model convergence, and there was significant overlap between district and SES (*r* = 0.58, *p* < 0.001). For longitudinal analyses, we present both unweighted (complete case data) and sample design and attrition adjusted estimates for clarity, but our primary results are those taken from the adjusted analyses.

Our sample included five sibling pairs and to ensure that this had no bearing on our findings all analyses were repeated randomly excluding one sibling. This did not influence either the weighted prevalence estimate or longitudinal stability of diagnoses.

## Results

### Descriptive statistics

The descriptive statistics for the full sample (*n* = 277) at Wave 1, and an attrition analysis for those who took part at Waves 1 and 3 vs. those who took part at Wave 1 only can be found in Table 1.

**Table 1:** Demographics for the full QUEST sample at Wave 1 and attrition analysis

	Wave 1 full sample (*N* = 277)	Completed Wave 1 and Wave 3 (*N* = 214)	Sample differences
District (% Lewisham/Bromley)	41/59	40/60	*p* = 0.81
Parental employment (% unemployed) (*n* = 267)	29	27	*p* = 0.57
Child ethnicity (% white yes/no^a^) (*n* = 268)	51/49	51/49	*p* = 0.99
Child sex (% male)	82	83	*p* = 0.65
Child SCQ	20.06 (7.43; 1–37)	20.11 (7.54; 1–37)	*p* = 0.93
Child IQ (*n* = 273)	72.58 (26.53; 19–129)	73.94 (26.52; 19–129)	*p* = 0.46
Child DBC	71.16 (29.61; 6–141)	71.28 (30.46; 6–141)	*p* = 0.95

*SCQ* Social Communication Questionnaire, *DBC* Developmental Behaviour Checklist Total Problems Score

^a^This included Black Caribbean (14%), Black African (15%), mixed ethnicity (10%), Pakistani (< 1%), Indian (< 1%), Bangladeshi (< 1%), Chinese (< 1%) and other (8%)

### Weighted prevalence of co-occurring psychiatric diagnoses in adolescence

The overall weighted 3-month point prevalence for any DSM-5 disorder was found to be 71.1%. The prevalence of emotional disorder was found to be 62.3%, which included a 10.7% prevalence of major depression and 51.4% for any anxiety disorder. Of the anxiety disorders, generalised anxiety disorder (GAD; 31.9%), social phobia (22.9%) and specific phobia (20.8%) were most prevalent. Obsessive–compulsive disorder (OCD) was present in 29.6%. Anxiety disorders commonly overlapped with 37.5% of the sample having more than two anxiety diagnoses and 18% having more than three. ADHD was present in in 28.6%, and for behavioural disorders; oppositional defiant disorder (ODD) in 12.5% and conduct disorder in 6.7%. The prevalence estimates for the remaining DSM-5 diagnoses and 95% confidence intervals are presented alongside the estimates from Wave 1 (Salazar et al. [20]) in Table 2. Prevalence rates for the broader overarching categories of emotional disorders, behavioural disorders, ADHD were also calculated split by sex (See Supplementary Materials). Females were found to have higher rates of emotional disorders and ADHD, whilst having lower rates of behavioural disorders. Although as seen below these are not considered to be significant differences. Unweighted prevalence estimates for these categories and the overlap between them can also be found in the Supplementary Materials.

**Table 2:** Weighted prevalence estimates in adolescence and comparison to childhood

DSM-5 diagnosis	Prevalence estimates age 13–17 years (95% CI) (*N* = 277)	Estimate age 4–10 years (95% CI) Salazar et al. [20]
Any DSM-5 disorder	71.1% (59.7–82.6)	90.5% (84.2–96.7)
Any emotional disorder	62.3% (50.3–74.4)	80.0% (70.7–87.0)
Major depression	10.7% (2.5–18.8)	14.6% (6.0–23.2)
Any anxiety disorder	52.2% (39.4–65.1)	78.9% (70.7–87.0)
Separation anxiety disorder	8.2% (1.6–14.8)	18.6% (9.4–27.8)
Generalised anxiety disorder	31.9% (21.6–42.3)	66.5% (57.0–76.0)
Specific phobia	20.8% (10.6–30.9)	15.1% (6.2–24.0)
Social phobia	22.9% (12.2–33.8)	11.4% (1.7–21.0)
Agoraphobia	9.4% (2.2–16.6)	18.0% (9.2–26.7)
Panic disorder (note with frequency)	4.6% (0.0–11.3)	3.1% (0–7.5)
Obsessive–compulsive and related disorders	–	–
Obsessive compulsive disorder	28.6% (16.4–40.7)	–
Trichotillomania	6.7% (0.0–13.3)	–
Any ADHD	28.6% (17.3–39.9)	59.1% (47.3–70.9)
Combined type	4.5% (0.0–10.0)	–
Predominantly inattentive	19.5% (10.1–28.9)	–
Predominantly hyperactive	5.1% (0.0–9.7)	–
Any behavioural disorder^a^	14.9% (5.7–24.1)	
Oppositional defiant disorder	14.9% (5.7–24.1)	28.7 (18.2 – 39.2)
Conduct disorder	6.7% (0.0–13.9)	2.0 (0.0–6.1)
Tourette’s disorder	4.2% (0.0–12.9)	
Any vocal/motor tics	11.4% (4.0–18.8)	17.4% (8.1–26.7)
Encopresis	5.8% (0.0–11.2)	1.9% (0.4–4.3)
Enuresis	8.7% (2.2–15.4)	13.5% (5.5–21.5)

^a^All participants with conduct disorder also met criteria for ODD

### Predictors of co-occurring psychiatric diagnoses

Diagnoses were collapsed into their overarching categories of emotional disorders, behavioural disorders, and ADHD, and sex and FSIQ (measured at Wave (1) were tested as predictors of disorder status at Wave 3. There were no significant relationships between sex, FSIQ and the presence of diagnostic status across all disorder categories (see Supplementary Materials).

### Longitudinal stability of psychiatric diagnoses from childhood to adolescence

**Fig. 2:**
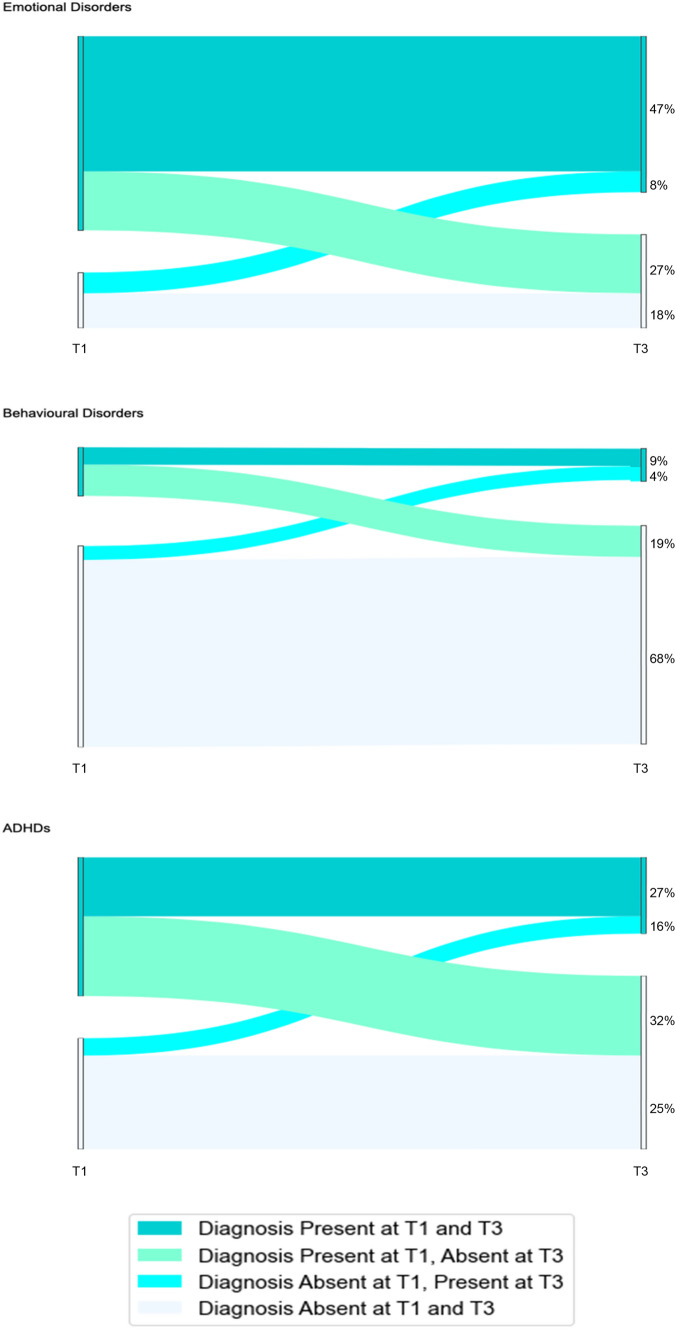
Stability and change within disorder categories from childhood to adolescence in autistic youth

The analyses indicated substantial stability in presence of weighted diagnoses from childhood to adolescence; see Fig. 2 for a visualisation of the proportion of young people who maintained or changed their diagnostic status from childhood to adolescence. Results indicated a high probability that those with an emotional disorder in childhood will continue to meet diagnostic criteria in adolescence (OR = 8.38; 95% CI 1.4–50.4; *p* = 0.02). It was estimated that 47% (95% CI 36–58%) of the sample had an emotional disorder in childhood and retained this into adolescence. In contrast, only 8% (95% CI 4–17%) were estimated to develop a new emotional disorder between child and adolescent assessments. When treated separately from other emotional disorders depression was not found to be significantly stable from childhood to adolescence (OR = 7.14; 95% CI 0.48–105.4; *p* = 0.15), but this should be interpreted with caution as the low numbers of individuals with depression mean these analyses are likely underpowered.

Results indicated a high probability that those with a behavioural disorder in childhood would also to meet diagnostic criteria in adolescence (OR = 27.4; 95% CI 3.5–212.4;* p* < 0.01). Most participants never met diagnostic criteria for a DSM-5 behavioural disorder but 9% (95% CI 4–18%) had a diagnosis which was stable from childhood to adolescence and 19% (95% CI 13–25%) met criteria in childhood but lost the diagnosis in adolescence. This leaves a small proportion of youth, 4% (95% CI 1–10%), who developed a behavioural disorder between childhood and adolescence.

Whilst adolescent ADHD status was predicted by childhood ADHD status, when adjusted for study design and attrition this was not significant (OR = 2.8; 95% CI 0.3–20.4;* p* = 0.31). Whilst 27% (95% CI 18–37%) showed stability in ADHD diagnoses between childhood and adolescence, 32% (95% CI 23–44%) who had a childhood diagnosis no longer met diagnostic criteria in adolescence, and 16% (95% CI 10–24%) of the sample transitioned from no ADHD diagnosis to meeting diagnostic criteria by adolescence. To account for the possibility that non-significance was driven by some adolescents dropping just below diagnostic threshold the analyses were repeated reducing the symptom criteria from six, to five and then four. This did not influence our findings (see Supplementary Table 1).

The full results based on both those adjusted for sample design and attrition and unadjusted (complete case data) are presented in Table 3.

**Table 3:** The diagnostic stability of disorder categories from childhood to adolescence

	Wave 3 diagnostic status in same disorder category			
	Unadjusted (complete case data) (*N* = 77)		Adjusted for sample design and attrition (*N* = 277)	
	Log Odds	Odds ratio	Log Odds	Odds ratio
Wave 1 Emotional disorder	1.34 (95% CI 0.18–2.50), *p* = 0.024	3.82 (95% CI 1.20–12.21)	2.13 (95% CI 0.33–3.92), *p* = 0.020	8.38 (95% CI 1.39–50.37)
Wave 1 behavioral disorder	2.01 (95% CI 0.52–3.51), *p* = 0.008	7.50 (95% CI 1.69–33.35)	3.31 (95% CI 1.26–5.36), *p* = 0.002	27.40 (95% CI 3.54–212.41)
Wave 1 ADHD	1.38 (95% CI 0.24–2.53), *p* = 0.017	3.99 (95% CI 1.27–12.50)	1.03 (95% CI − 96.05–3.02), *p* = 0.311	2.79 (95% CI 0.38–20.38)

## Discussion

Current findings are consistent with previous studies in autistic children and adolescents that report high rates of co-occurring psychiatric diagnosis. To our knowledge, this is the first study of autistic youth to report prevalence longitudinally using comparable gold-standard diagnostic instruments, enabling the investigation of diagnostic stability between childhood and adolescence. By using a community-based sampling strategy, paired with a weighted statistical approach, we can present representative prevalence rates of psychiatric disorders in autistic adolescents which are more generalisable than most previous studies.

We found that 71.1% of autistic adolescents experience at least one co-occurring DSM-5 disorder. The rate of any emotional disorder of 62.3%, with 51% experiencing at least one anxiety disorder, is broadly consistent with estimates from comparable population-based studies [2]. As is commonly reported, GAD and social phobia were the most prevalent anxiety diagnoses for autistic adolescents [18]. The 10.7% prevalence rate for major depression is substantially higher than other estimates from community-based samples [2], with the difference likely accounted for by age (10–14 years versus 13–17 years in the current study). However, this rate is consistent with meta-analytic evidence of a prevalence of 11% across all age groups in autism [3]. This is double the expected prevalence of around 5% in the typically developing youth population [31]. The rate of depression at wave 1 of the study (14.6%) was especially high and should be interpreted with caution [20]. Rather than this indicating a reduction in the prevalence of depression from childhood to adolescents this may in part reflect limitations of PAPA in discriminating depression from some symptoms of autism (flattened affect, social withdrawal, etc.) which may be a particular challenge in 4–10-year-olds who may have greater difficulty labelling internal states and communicating these to their parents, when compared to the adolescent period.

The rates of OCD were found to be high (28.6%), even when compared to previous studies that have identified OCD as a prominent issue in autism [32], although it should be noted that rates of OCD of up to 37% have been reported [33]. Assessing OCD in the context of autism often raises issues around diagnostic overshadowing or the potential to confuse of autistic stereotypies or special interests as obsessions and/or compulsions [34]. It is also important to note that some changes to the OCD criteria (e.g., removal of criteria around insight) in the DSM-5 may increase the number of autistic youths meeting diagnostic thresholds. In this study, both factors were dealt with by consideration at a symptom level ensuring accurate coding as either a behaviour associated with autism or OCD. Regardless, this finding should be interpreted cautiously, and possible OCD symptoms explored in detail when working with autistic youth to best inform the most appropriate interventions.

The prevalence rate of 28.7% for ADHD is consistent with previous studies and meta-analyses in autism populations [3]. This is compared to a prevalence rate in neurotypical youth of between 2 and 7% [35], with a peak in prevalence being found around the age of 9 years [36]. The ADHD prevalence rate was driven primarily by the predominantly inattentive type (Table 2), with the rate for the combined- and predominantly hyperactive type being more consistent with the typically developing youth population. This finding was also found in the childhood wave of the study [20], suggesting this pattern is consistent over-time and may indicate the high prevalence of ADHD in autism is in part driven by underlying cognitive processes associated with inattention. Evidence suggests that symptoms of inattention and autism, as well as broader difficulties in executive function tend to load together and may have a common neuro-genetic aetiology [37].

As expected, the rates of ODD in the sample are reduced from those reported in childhood but remain high compared to prevalence rates of between 1.6 and 6.2% in neurotypical adolescents [38]. Rates of conduct disorder were lower but did increase from 2 to 6.7% from childhood to adolescence. It is relevant to note that all those who met DSM-5 criteria for conduct disorder also met criteria for ODD.

We found that neither sex nor intellectual ability (measured in childhood) predicted diagnostic status in adolescence. These null findings are in line with previous work which has found few established risk factors predict psychiatric diagnosis in autistic youth [2]. At wave 1 of this study, higher intellectual ability was significantly associated with risk for any anxiety disorder [20], which is generally supported in the literature [17]. However, a limitation is that intellectual ability was measured in childhood and, therefore, did not account for any change in FSIQ between childhood and adolescence. We also found that sex did not significantly predict prevalence across any of the diagnostic categories. This contrasts with the previous finding of a greater prevalence of emotional disorders in autistic females [11, 12]. With regards ADHD and behavioural disorders, the reduction in prevalence in adolescence may account for the lack of replication with the associations found at Wave 1 of the current study. Whilst it is possible that the current study was underpowered to detect the effects of sex and IQ, based, for example, on the reported sex differences in the rates of emotional disorders and ADHD in neurotypical children, our current sample should have been of adequate size to detect significant effects. This study was limited in the range of factors measured which may have related to prevalence of co-occurring psychiatric conditions. Future research would benefit from further consideration of wider environmental and contextual factors, such as peer relationships, access to intervention, age at diagnosis of autism or family factors that may influence outcomes, as well as investigating interactions with individual characteristics, to investigate person-environment fit [39].

### Diagnostic stability between childhood and adolescence

We found high levels of stability in emotional disorders, with nearly half of the sample who met diagnostic criteria for an emotional disorder in childhood continuing to meet diagnostic threshold in adolescence. The stability of emotional disorders is consistent with other population-based studies in autistic youth examining change in symptoms between adolescence and adulthood [8]. Although difficult to make comparisons due to the limited literature and different sampling strategies, the stability of emotional disorders in autistic youth appears to be greater than in neurotypical youth; with the current finding of an OR of 8.38 being substantially higher than ~ 3 reported by Copeland et al. [6].

Behavioural disorders were also found to be stable but being driven more strongly by most young people not meeting diagnostic criteria in either childhood or adolescence, and a smaller group (9%) with a persistent diagnosis. Overall, 19% of participants no longer met diagnostic criteria for a behavioural disorder by adolescence. This is consistent with the literature reporting declining trajectories of behavioural symptoms over-time in autistic youth [40].

Despite relatively strong prediction of adolescent ADHD based on childhood diagnosis (OR = 2.79), this did not reach significance in our adjusted analysis due to wide confidence intervals. Whilst around 27% of the sample had stable diagnoses from childhood to adolescence, a similar proportion (32%) no longer met diagnostic criteria. This resulted in a drop in the prevalence of ADHD, which is in part consistent based on evidence from studies in both autism [41] and non-autistic samples [38]. It is also notable that 16% of the sample met diagnostic criteria for ADHD in adolescence who did not have a childhood diagnosis. One possibility is that it is harder to distinguish the symptoms of autism and ADHD in younger children leading to reduced stability when compared to emotional or behavioural diagnoses. In routine clinical practice, direct observation of the child is recommended to distinguish between symptoms of autism and ADHD, which not part of the research diagnostic assessment used in this study. This may also be related to the high rates of the predominantly inattentive sub-type of ADHD within this sample, whereby the impact of the cognitive features of ADHD may become more pronounced as educational and social demands increase in adolescence. These findings highlight the importance of assessing for ADHD in adolescents who may not have met diagnostic criteria in childhood, but whose symptoms may nevertheless be having a significant impact.

The stability of emotional and behavioural disorders suggests that by early- to mid-childhood, it may be possible to predict who will continue to require clinical support through to the adolescent period. This highlights both the importance of intervention/prevention in early childhood, and the need for continued monitoring and support during adolescence, where psychiatric morbidity remains high.

Strengths of the current work include use of a community-based longitudinal sample of autistic youth, detailed diagnostic assessments of psychopathology in both childhood and adolescence, and the over-sampling of females to have power to account for sex in prevalence estimates. There are several limitations that should also be considered. Whilst the sample is community-based, it was selected from two urban/sub-urban regions both in South London and, therefore, may not be representative of all autistic young people. In addition, this sample was selected from those with existing autism diagnoses and accessing clinical services. This may have inflated our prevalence estimates as access to services may be associated with higher levels of emotional and behavioural difficulties. Similarly, there is evidence to suggest that those young people who received their diagnosis of autism earlier in childhood (as was the case in this study), have a trajectory of experiencing greater mental health and behavioural difficulties compared to those diagnosed later in childhood [42]. In addition, whilst accounted for in-part by the statistical approach, the over-sampling of females may have inflated the prevalence estimates of some diagnoses more than others. However, our findings were mostly consistent with similar studies with population representative sampling and a male: female ratio more typical of the literature [2]. Finally, to maximise the clinical utility of these findings, we chose to use the most up to date diagnostic classifications at each wave switching from DSM-IV to DSM-5. It is possible this influenced our results due to changes in some diagnoses, for example, the removal and updating of several items from the OCD criteria.

### Supplementary Information

Below is the link to the electronic supplementary material.

Supplementary file1 (DOCX 213 KB)
