# Supplementary material for: Psychiatric conditions in autistic adolescents: longitudinal stability from childhood and associated risk factors
Source: Eur Child Adolesc Psychiatry. 2022 Aug 17;32(11):2197–208. doi: 10.1007/s00787-022-02065-9 (PMC10576662; doi:10.1007/s00787-022-02065-9)
Supplement: Supplementary file 1 — Supplementary file1 (DOCX 213 KB) [file 787_2022_2065_MOESM1_ESM.docx]

## **Supplementary Materials**

*Supplementary Figure 1. Latent Class Transition model* *Used to Estimate Longitudinal Prevalence and Predictors*

**
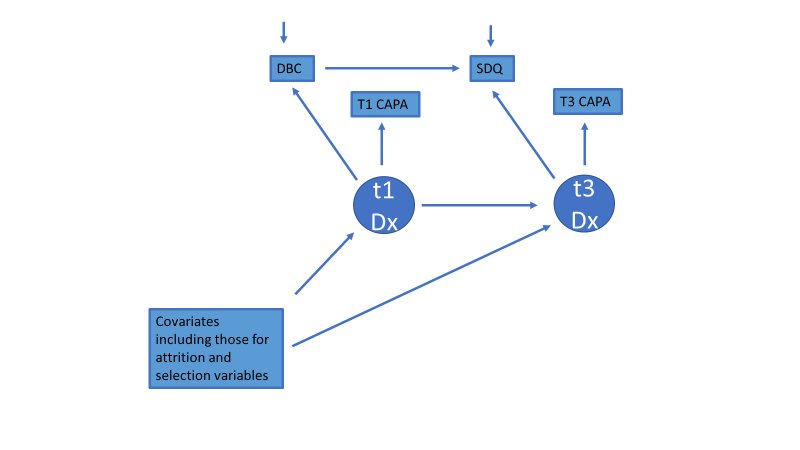
**

DBC = Developmental Behaviour Checklist; SDQ = Strengths and Difficuties Questionnaire; CAPA= Child & Adolescent Psychiatric Assessment.

*Supplementary Figure 2. Un-weighted Frequency of Emotional, Behavioural and ADHD Diagnosis and their overlap (n=72)*

*
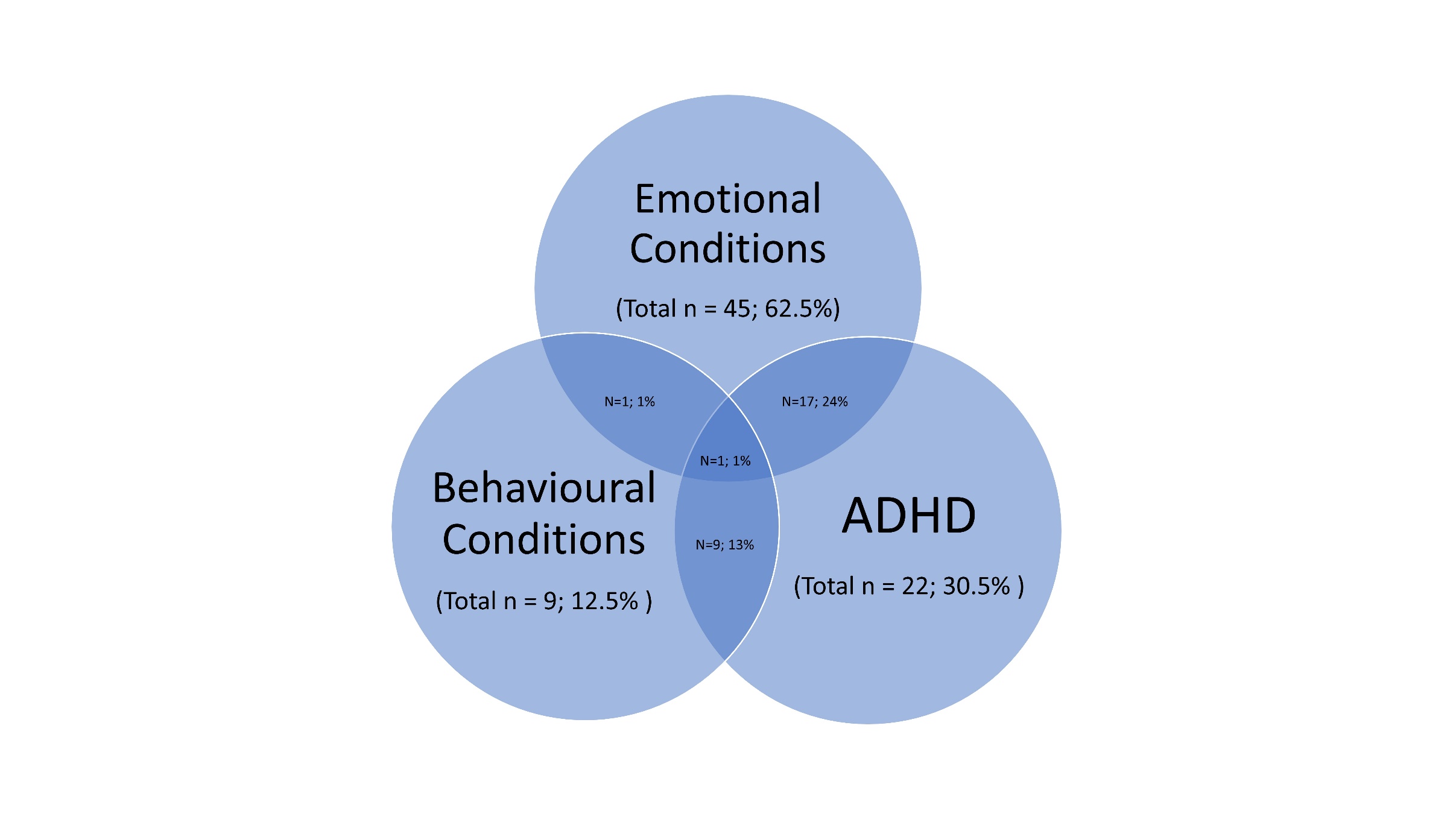
*

*Supplementary Table 1. Wave 3 weighted prevalence across the main diagnostic categories by gender*

| DSM-5 Diagnosis | Prevalence estimates in Males (95% CI) (N=232) | Prevalence estimates in Females (95% CI) (N=50) |
| --- | --- | --- |
| **Any emotional disorder** | 54.4% (41.5-67.3) | 64.8% (49.5-80.1) |
| **Any ADHD** | 27.7% (16.1-39.4) | 38.6% (24.4-52.7) |
| **Any behavioural disorder** | 14.5% (4.9-24.0) | 8.4% (0.09-15.9) |

*Supplementary Table 2. Diagnostic Stability of ADHD Diagnosis as Function of Stringency of DSM-5 Criteria in Adolescence*

|  | Unadjusted (complete case data) | | Adjusted for sample design and attrition | |
| --- | --- | --- | --- | --- |
|  | Log Odds | Odds Ratio | Log Odds | Odds Ratio |
| Full criteria: 6+ symptoms, w/ onset <12years | 1.38 (95% CI .24 -2.53), p=.017 | 3.99 (95% CI 1.27-12.50) | 1.03 (95% CI -.96.05-3.02), p=.311 | 2.79 (95% CI 38-20.38) |
| 5+ symptoms, w/ onset <12years | 2.01 (95% CI .52-3.51), p=.008 | 7.50 (95% CI 1.69-33.35) | 1.4 (95% CI -.76.26-3.57), p=.204 | 4.07 (95% CI .47-35.55) |
| 4+ symptoms, w/ onset <12years | 1.29 (95% CI .28-2.31), p=.007 | 3.67 (95% CI 1.33-10.10) | 1.36 (95% CI -.35-5.21), p=.647 | 0.31 (95% CI -1.02-1.65) |

*Supplementary Table 3. Participant characteristics predicting diagnostic outcome*

|  | Emotional Disorder | Behavioral Disorder | ADHD |
| --- | --- | --- | --- |
| Sex | b=.51, p=.33 | b=-.50, p=.53 | b=.41, p=.44 |
| Wave 1 FSIQ | b=-.01, p=.78 | b=.03, p=.15 | b=-.01, p=.48 |
